# Supplementary material for: TUT‐DIS3L2 is a mammalian surveillance pathway for aberrant structured non‐coding RNAs
Source: EMBO J. 2016 Sep 19;35(20):2179–91. doi: 10.15252/embj.201694857 (PMC5069555; doi:10.15252/embj.201694857)
Supplement: Supplementary file 1 — Appendix [file EMBJ-35-2179-s001.pdf]

APPENDIX

TUT-DIS3L2 is a mammalian surveillance pathway for aberrant structured noncoding RNAs

Dmytro Ustianenko, Josef Pasulka, Zuzana Feketova, Lukas Bednarik, Dagmar Zigackova, Andrea Fortova, Mihaela Zavolan, Stepanka Vanacova

TABLE OF CONTENTS

Table S1
Table S2
Supplementary Methods
Supplementary References

Table S1 Table illustrating that TDS targets 3' extended uridylated forms of snRNAs. The table shows CLIPed snRNAs and coverage of regions downstream of snRNA mature 3' termini (three_prime_overhang). The last column is a graphical representation of the U+ read coverage.

transcript_name	transcript_id	transcript_biotype	sum_uri_reads	three_prime_overhang	reg_id	coverage_image
RNU5B-1-201	ENST00000363286	snRNA	5220	266	15:65304686-65305058:+	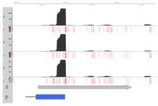
U11.1-201	ENST00000387069	snRNA	4920	82	1:28648599-28648815:+	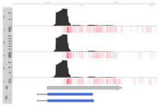
U12.1-201	ENST00000362512	snRNA	3150	299	22:42615260-42615692:+	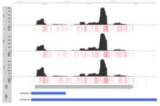
RNVU1-7-201	ENST00000383858	snRNA	2910	223	1:148038530-148038841:-	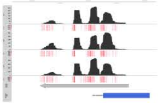
U1.29-201	ENST00000615842	snRNA	2700	226	1:146376910-146377196:+	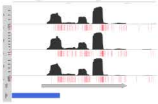
RNU4ATAC-201	ENST00000580972	snRNA	2650	341	2:121530899-121531348:+	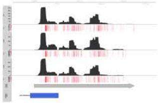
RNU5A-1-201	ENST00000362698	snRNA	2260	177	15:65296056-65296343:+	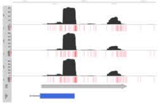
U1.22-201	ENST00000605806	snRNA	2030	103	1:145465514-145465760:-	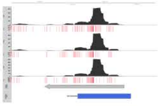
RNU1-27P-201	ENST00000383869	snRNA	1240	175	14:34546782-34547052:+	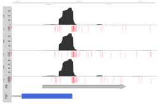
RNU2-2P-201	ENST00000410396	snRNA	1180	293	11:62841326-62841746:-	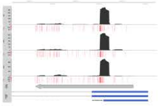
RNU5F-1-201	ENST00000362507	snRNA	911	342	1:44721444-44721901:-	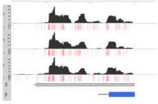
RNU5A-8P-201	ENST00000364102	snRNA	799	35	1:210374119-210374270:-	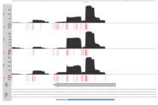
RNU5D-1-201	ENST00000363299	snRNA	759	28	1:44731027-44731142:-	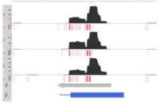
RNU1-28P-201	ENST00000383861	snRNA	743	21	14:34556205-34556272:-	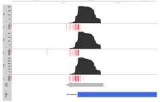
RNU5E-6P-201	ENST00000365574	snRNA	704	169	1:44819714-44819988:-	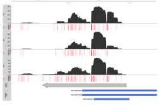

transcript_name	transcript_id	transcript_biotype	sum_uri_reads	three_prime_overhang	reg_id	coverage_image
U1.32-201	ENST00000622285	snRNA	588	57	1:148522598-148522822:+	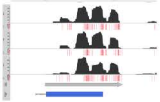
RNU6-1-201	ENST00000383898	snRNA	519	46	15:67839893-67839980:-	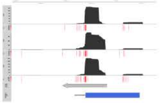
RNU1-2-201	ENST00000384278	snRNA	500	161	1:16896018-16896304:+	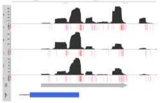
RNU1-4-201	ENST00000384659	snRNA	476	157	1:16740602-16740836:+	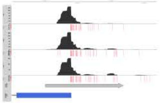
RNVU1-6-201	ENST00000364688	snRNA	353	23	1:146052058-146052226:-	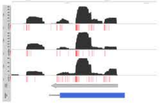
U2.3-201	ENST00000619225	snRNA	321	22	17:43290269-43290345:-	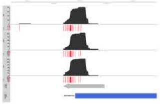
RNU4-2-201	ENST00000365668	snRNA	319	74	12:120291689-120291895:-	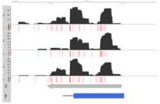
RNU5E-4P-201	ENST00000364931	snRNA	281	110	1:11909698-11909903:-	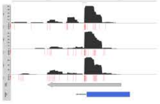
RNU5E-1-201	ENST00000362477	snRNA	232	53	1:11908150-11908324:+	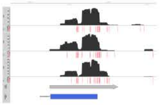
RNVU1-10-201	ENST00000384610	snRNA	217	48	1:148362322-148362505:-	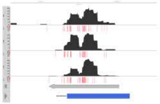
RNU1-3-201	ENST00000384782	snRNA	98	102	1:16666683-16666855:-	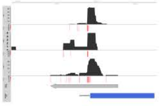
RNU6-1035P-201	ENST00000384477	snRNA	80	21	9:81725222-81725269:-	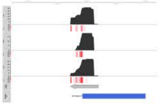
RNU4-1-201	ENST00000363925	snRNA	79	40	12:120293057-120293205:-	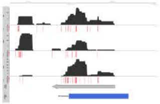
RNU1-92P-201	ENST00000517017	snRNA	21	57	1:143720453-143720547:-	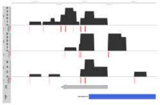
RNU6-2-201	ENST00000384627	snRNA	21	30	19:1021612-1021658:+	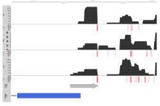
RNU1-85P-201	ENST00000364127	snRNA	13	60	17:58679467-58679537:-	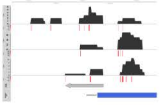

Table S2. The list of oligonucleotides used for RT-PCR and northern blot analyses

Primer	Sequence (5'-3')
trna136-ValAAC 5'	TAACCACTACACTACGGAAAC
trna136-ValAAC 3'	GTTTCCGCCCGGTTTCGAACC
trna141-LeuCAA 5'	AGACCACTCGGCCATCCTGAC
trna141-LeuCAA 3'	CCCCAAACCTGGGAAGTAGCA
trna149-LysTTT 5'	TACCGACTGAGCTACCCAGGC
trna149-LysTTT 3'	ACTTGAACCCTGGACCCTCAG
RT-CLIP2	AGACGTGTGCTCTTCCGATCT
RT-CLIP2-2U	AGACGTGTGCTCTTCCGATCTAA
U12extF	ACCTTATTCACGCCTAAAAA
U12F	TGCCTTAAACTTATGAGTAA
U5FextF	GGTGTTTCATGCTTTGGGAGGTTG
U5FF	ATCTCTGGTTTCTCTTCATA
OAT F	GGCGTCACTGTTGCGCTTCATAGAC
RPS12 F	GAGTCGCGCGGAGGCGGAGGC
RPL12 F	TTCCGGCCTCTCGGCTTTCG
RN7SL1 for	ATCGGGTGTCCGCACTAA
RN7SL1 rev	TCCGTTTCCGACCTGGGCCGG
5SrRNA for	TACGGCCATACCACCCTGAA
5SrRNA rev	GCGGTCTCCCATCCAAGTAC

Supplementary Methods

Mapping of the CLIP reads on human genome

All DIS3L2 mutant CLIP replicates were processed in parallel. Primary mapping on the reference genome GRCh37/hg19 was done with the Segemehl software (Hoffmann, Otto et al., 2009). The histogram of mononucleotide tail lengths revealed that tail lengths over 3 nucleotides were very rare except for oligo(U) (Fig 1B). Therefore only the reads containing at least four terminal uridines were denoted as uridylated and selected for further analyses. Fifty percent of the reads that did not map to the genome initially contained four or more 3′-terminal uridines, and 7% of them mapped to the genome after U-tail removal under strict mapping conditions described in Statistics below (Fig 1C, EV1A, EV1B). All reads that mapped uniquely to the genome either in the first or second round of mapping were compared with the reference genome for the presence of untemplated poly(T) tails. All reads containing at least 4 extra untemplated Ts at the 3' end (allowing one mismatch) were considered "uridylated".

Because rDNA is not included in the assembly of the human genome, the rRNA transcripts of PolI, would be excluded from the analysis. We therefore used an assembly of the rDNA gene cluster from Genbank (http://www.metalife.com/Genbank/U13369_555853) as an artificial chromosome that allows us to annotate rRNA-derived reads.

Statistics

To select only the most significant DIS3L2 targets, we divided the genome into 50 nt regions and analyzed these windows as follows. We calculated the coverage of each window by reads and discarded the windows where the sum of reads mapped in all tree replicates of the experiment was less than 20. We then separated the reads mapping in each window into those that had and those that did not show evidence of uridylation. Let us call the number of uridylated reads in window $\boldsymbol{i}$ u_i and the number of reads that were not uridylated and mapped in this window n_i Let $\boldsymbol{p_i}$ be the underlying probability of uridylation in a region $\boldsymbol{i}$, then the probability of the data is

$$P(u_i|\, p_i, u_i + n_i) \ = p_i^{u_i}(1 - p_i)^{n_i}$$

From Bayes theorem we infer the posterior probability of $\boldsymbol{p_i}$:

$$P(p_i|\, u_i \ + \ n_i) \ = \ \frac{(u_i + n_i + 1)!}{u_i!\ n_i!}\ p_i^{u_i}\ (1 - p_i)^{n_i}$$

The probability of p_i beeing greater than threshold value $\boldsymbol{A}$ is proportional to incomplete beta function:

$$\int_A^1 dp\ P(p_i \mid u_i, u_i + n_i) \ = \ 1 \ - \ \frac{(u_i + n_i + 1)!}{u_i!\ n_i!} \ Beta(A, 1 + u_i, 1 + n_i).$$

This calculation was done for each of the three replicates. The total probability $\boldsymbol{p}$ of $\boldsymbol{p_i} \geq \boldsymbol{A}$ in each sample is a simple product of all probabilities $\boldsymbol{p_i}$. In our analysis, we selected the windows which had at least 0.95 probability that the frequency of uridylation was greater than 0.25.

To increase the confidence in the uridylated regions we repeated our approach selecting windows with untemplated poly(A), poly(C) and poly(G) tails. The final statistics is shown in Fig EV1A. The number of selected significant regions is in the following table.

**Tail type**	**Number of selected significant 50 nt windows (95% confidence that uridylation frequency is at least 0.25)**
poly(A)	3
poly(C)	0
poly(G)	1
poly(T)	2991

Metagene analysis of the position of U+ reads and RNAP II position around mRNA TSS

The Chip-seq data used in this publication have been downloaded from National Center for Biotechnology Information's Gene Expression Omnibus under accession numbers GSM935534 (Yale_ChipSeq_HEK293_Pol2_std) and GSM945288 (UW_ChipSeq_HEK293_H3K4me3). We have identified a list of TSS with a region covered by uridylated reads 1kb upstream or downstream, respectively. For this purpose, we selected only those regions having at least 10 uridylated reads in sum over all three replicates. The coverage densities for all data were calculated separately for each TSS region from this list, all coverages were subsequently summarized and averaged over all replicates. Final coverage density represents a distribution of the reads around the listed TSS.

Expression and purification of TUTases and uridylation assay

DNA constructs for the expression human TUTases TUT4 (Minoda, Saeki et al., 2006) and TUT7 (Rissland, Mikulasova et al., 2007) were kind gifts from Dr. Yoshimura and Dr. Benecke, respectively. The gene for TUT6 was subcloned to the pCMV10 vector (Sigma-Aldrich) via restriction sites NotI and XbaI to obtain N-terminal fusion with 3xFlag tag. The

constructs were transfected to HEK293T-Rex cells. Forty eight hours after transfection cells were lysed in a buffer (50 mM Tris-HCl pH 8, 150 mM KCL, 0.5% TritonX100, 1 mM dithiothreitol, 1 mM PMSF, and 1 Complete Mini, EDTA-free protease inhibitor cocktail tablet (Roche). Cell lysates (TUT4 and TUT6) were applied on FLAG-magnetic beads (Sigma-Aldrich) and IgG FastFlow Sepharose (Amersham) (for TUTase 7). After one hour incubation with beads, bound complexes were extensively washed with Lysis buffer and Wash buffer (Lysis buffer containing 300 mM KCl). The *in vitro* uridylation reactions were performed in a total volume of 30 μl in 4 mM $MgCl_2$, 1 mM DTT, 0.25 mM UTP and 15 μl of immunopurified proteins on beads in Lysis buffer. The reaction mixture was incubated at 37 °C for 40 min. Reactions were terminated with one volume of formamide loading buffer (80% formamide, 0.1% bromphenol blue, 0.1% xylene cyanol, 5 mM EDTA). Reactions were resolved on denaturing 20% polyacrylamide gels containing 8 M urea. The radioactivity was exposed to phosphorimaging screen (FUJI) and scanned by phosphorimager FLA-9000 (FUJIFILM).

In vitro **degradation assay**

In vitro degradation assays were performed in 10 μl reaction volumes containing 10 mM Tris pH 8.0, 50 mM KCl, 5 mM $MgCl_{2,}$ 10 mM DTT (modified from (Ustianenko, Hrossova et al., 2013)). Typically, 150 nM of purified protein and 20 pmol of 5′-end labeled RNA substrate, were incubated at 37 °C for the times indicated. Reactions were terminated with one volume of formamide loading buffer (80% formamide, 0.1% bromphenol blue, 0.1% xylene cyanol, 5 mM EDTA). Reactions were resolved on denaturing 20% polyacrylamide gels containing 8 M urea. The radioactivity was exposed to phosphorimaging screen (FUJI) and scanned by phosphorimager FLA-9000 (FUJIFILM).

Supplementary References

Hoffmann S, Otto C, Kurtz S, Sharma CM, Khaitovich P, Vogel J, Stadler PF, Hackermuller J (2009) Fast mapping of short sequences with mismatches, insertions and deletions using index structures. PLoS computational biology 5: e1000502

Minoda Y, Saeki K, Aki D, Takaki H, Sanada T, Koga K, Kobayashi T, Takaesu G, Yoshimura A (2006) A novel Zinc finger protein, ZCCHC11, interacts with TIFA and modulates TLR signaling. Biochem Biophys Res Commun 344: 1023-30

Rissland O, S., Mikulasova A, Norbury C, J. (2007) Efficient polyuridylation by noncanonical poly(A) polymerases. Molecular and Cellular Biology 27: 3612-3624

Ustianenko D, Hrossova D, Potesil D, Chalupnikova K, Hrazdilova K, Pachernik J, Cetkovska K, Uldrijan S, Zdrahal Z, Vanacova S (2013) Mammalian DIS3L2 exoribonuclease targets the uridylated precursors of let-7 miRNAs. RNA 19: 1632-8
