# Supplementary material for: TUT‐DIS3L2 is a mammalian surveillance pathway for aberrant structured non‐coding RNAs
Source: EMBO J. 2016 Sep 19;35(20):2179–91. doi: 10.15252/embj.201694857 (PMC5069555; doi:10.15252/embj.201694857)
Supplement: Supplementary file 2 — Expanded View Figures PDF [file EMBJ-35-2179-s002.pdf]

Expanded View Figures

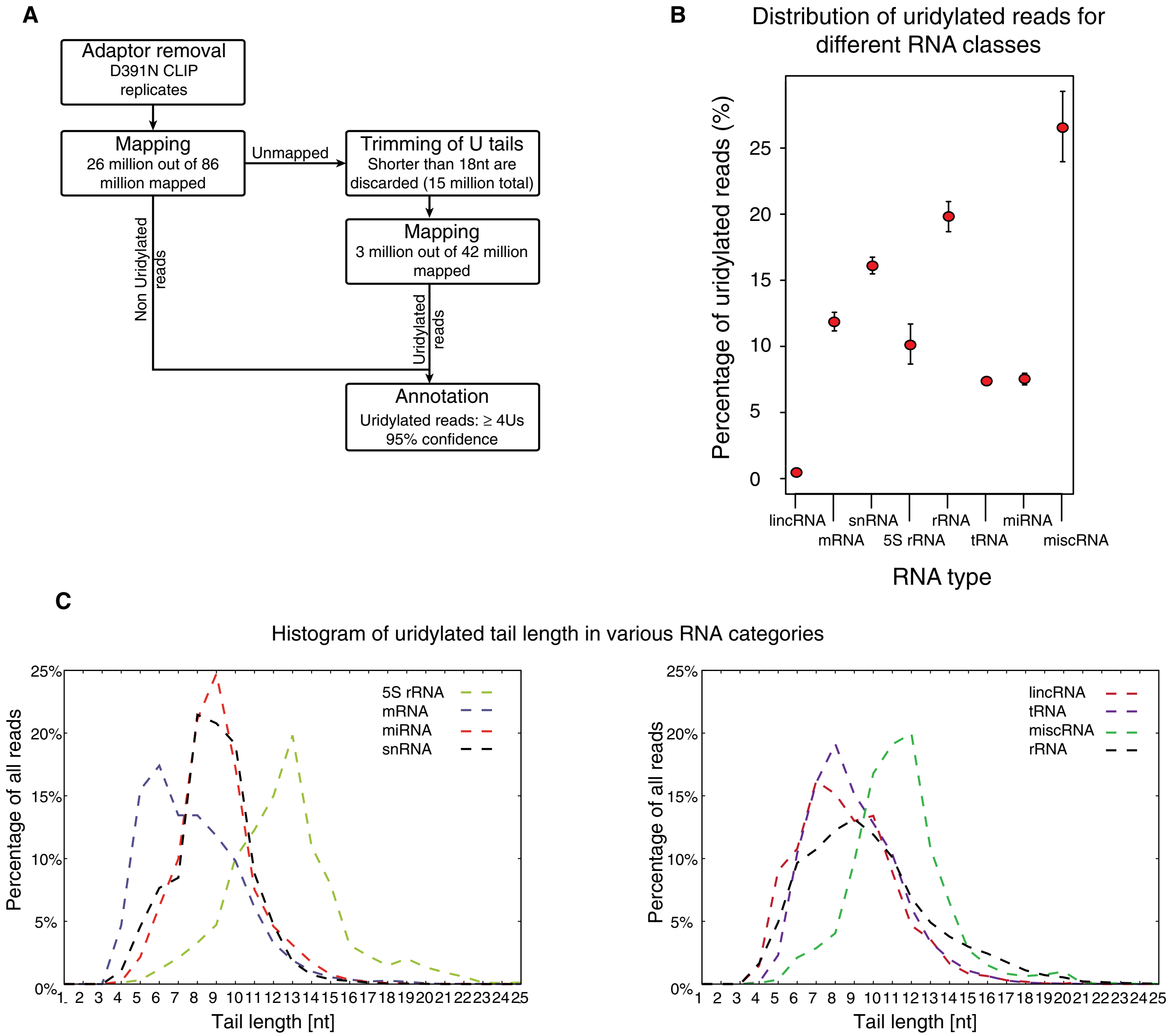

Figure EV1. Analysis of D391N DIS3L2 CLIP.

A Schematic representation of CLIP data analysis.

B Percentage of uridylated reads with at least 4Us for various functional categories of RNAs.

C Distribution of untemplated oligo(U) extension lengths for reads in various RNA annotation categories.

© 2016 The Authors

Figure EV2. Confirmation of DIS3L2 binding to aberrant forms of snRNAs by RIP followed by Sanger sequencing.

A The scheme showing the uridylated CLIP read coverage on mature snRNAs (in box below the graph) and regions downstream of their mature 3′ termini (line below the graph). The position of forward PCR primers annealing to the mature snRNAs (U12 F and U5F F) and to the regions downstream of the mature 3′ end (U12ext F and U5Fext F) is indicated at the bottom.

B RT–PCR analysis of U12 and U5 snRNAs co-precipitated with D391N DIS3L2. D391N-bound RNAs were ligated to 3′-terminal linker. Linker-specific primer was used for cDNA synthesis. PCR amplification was performed using the 3′ adaptor primer and primer specific to mature (U12 F and U5F F) or 3′-extended (U12ext F and U5Fext F) snRNAs, respectively. −RT ctrl is a PCR with RT reactions in which the reverse transcriptase was omitted, and PCR ctrl are controls where no cDNA was added. MW is molecular weight marker in base pairs (bp).

C Summary of sequencing results of snRNAs co-precipitated with D391N DIS3L2. Both mature and extended snRNA reads are shown, and gene-specific primer used for PCR is represented by the white box. The untemplated nucleotides are in red. Point mutations are in small letters, deletions indicated by −, for space constrains, the sequences were shortened, and the positions of interruption are indicated by dots. Wherever we identified identical sequences, they are indicated by number of repetitions in brackets on the right.

© 2016 The Authors

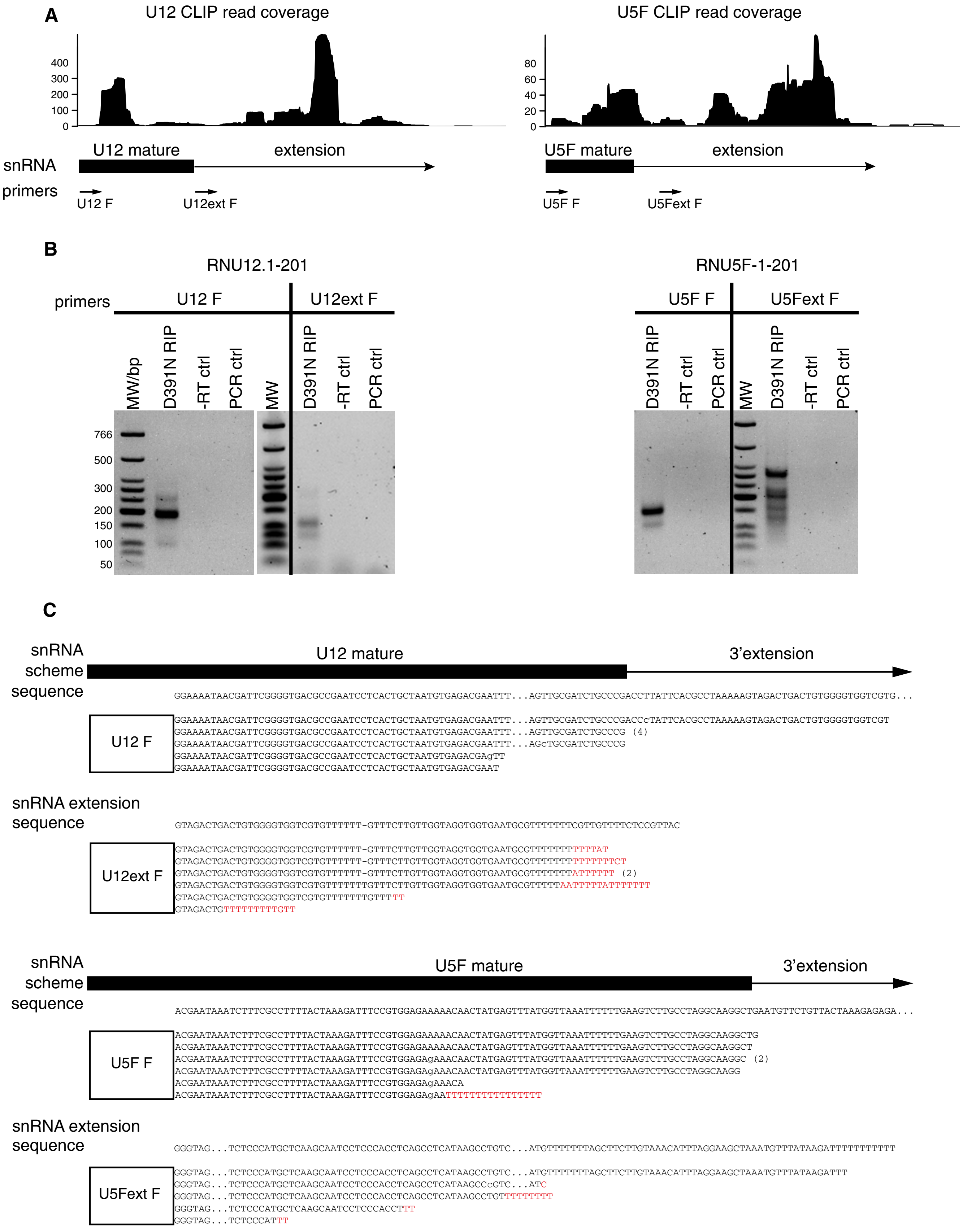

Figure EV2.

© 2016 The Authors

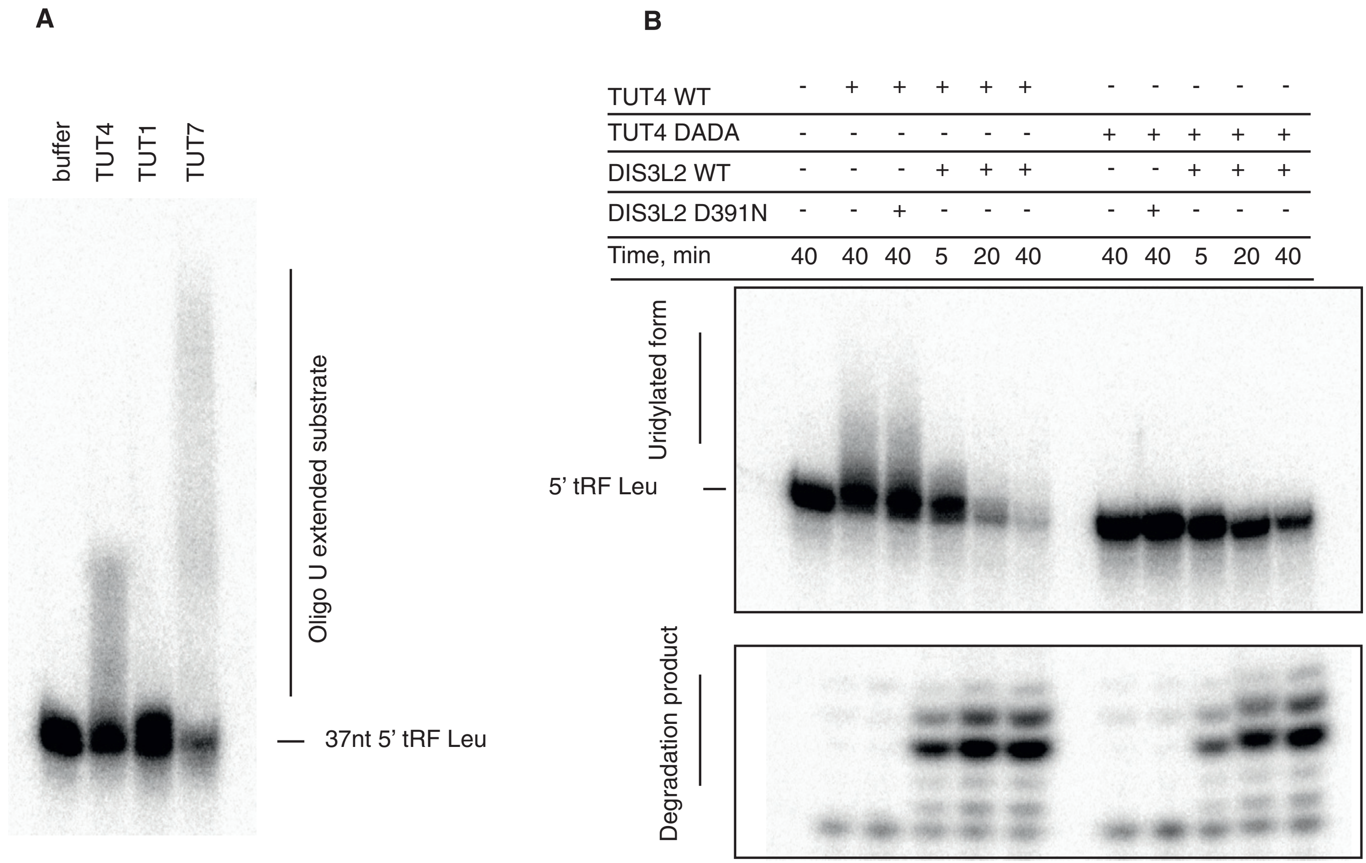

Figure EV3. TUT4 can uridylate tRNAs and TUT4 activity enhances tRNA degradation by DIS3L2.

A *In vitro* uridylation assay with TUTases 4, 6, and 7 purified from human cells and a Leu tRNA fragment (tRFLeu) as a template.

B Coupled, *in vitro* uridylation/degradation assay. 5′-end-labeled tRFLeu was incubated with wild-type (WT) or catalytically inactive (DADA) TUT4 for 40 min in the presence of 0.25 mM UTP. Subsequently, the WT or mutant DIS3L2 was added and the reactions were stopped after times indicated at the top of the panel. The RNAs were resolved on 20% denaturing acrylamide gel, and radioactivity was detected by autoradiography. The migration position of the input tRFLeu, the uridylated forms and the degradation products, is marked on the left side.

© 2016 The Authors

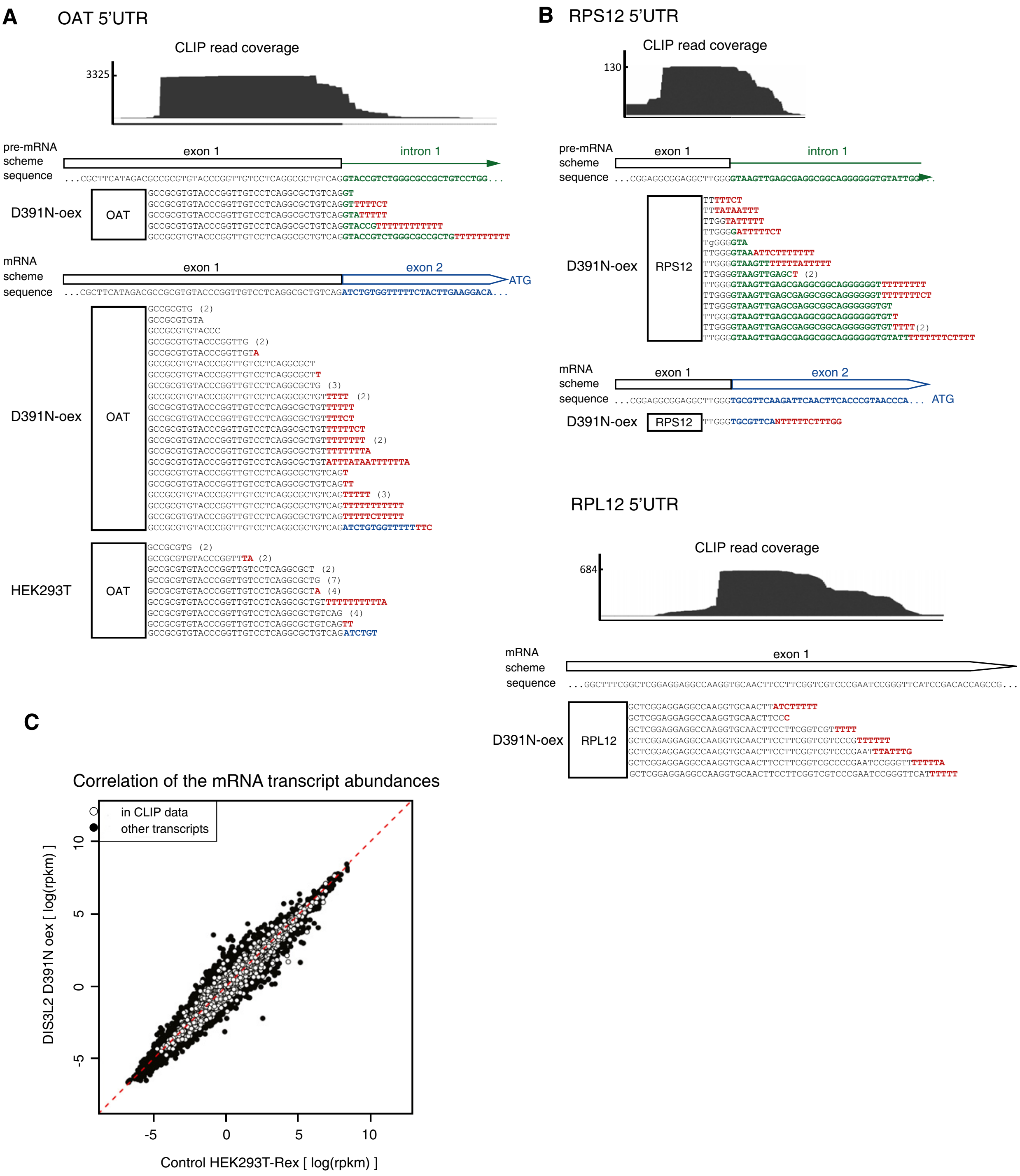

Figure EV4. DIS3L2 binds short uridylated transcripts derived from promoters of protein-coding genes (U+ 5′ mRFs).

A RT–PCR and sequencing analysis of the small RNA fraction isolated from the control HEK293T-Rex cell line and the cell line overexpressing D391N DIS3L2 (D391N-oex). Identification of uridylated transcripts of the *OAT* gene independent of DIS3L2 binding. The mature (spliced) first two exons are indicated below the graph together with the position of the translation start site (ATG). Gene-specific primer is represented by the white box. The untemplated 3′-terminal nucleotides detected by sequencing are shown in red. The numbers in brackets on the right indicate number of clones with identical sequences. Several clones from the D391N-oex cell line revealed uridylated unspliced transcripts that contained the first exon and part of intron 1 (schematically shown in the lower scheme).

B Identification of uridylated transcripts of *RPL12* and *RPS12* genes. The results are represented as described in (A).

C D391N DIS3L2 overexpression does not cause differential expression of protein-coding genes with DIS3L2-bound 5′ UTR fragments. Scatter plot of transcript abundances estimated from RNA-seq from the cell line overexpressing D391N DIS3L2 and control HEK293T-Rex cells. CLIPed transcripts are marked in white.

 © 2016 The Authors
