# Supplementary figures and images for: TUT‐DIS3L2 is a mammalian surveillance pathway for aberrant structured non‐coding RNAs
Source: EMBO J. 2016 Sep 19;35(20):2179–91. doi: 10.15252/embj.201694857 (PMC5069555; doi:10.15252/embj.201694857)

Figure 4D

OAT

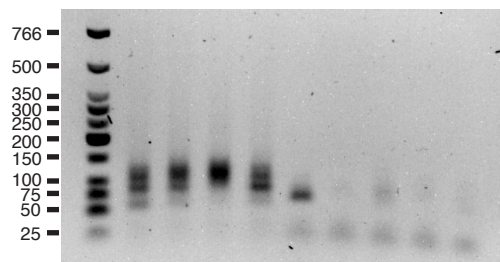

RPL12

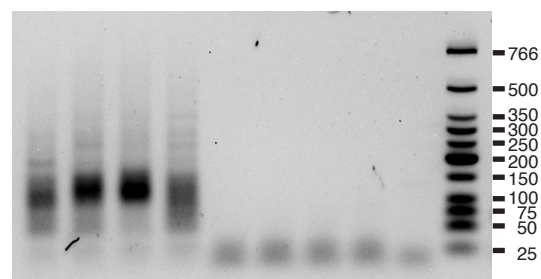

RNU12 ext.

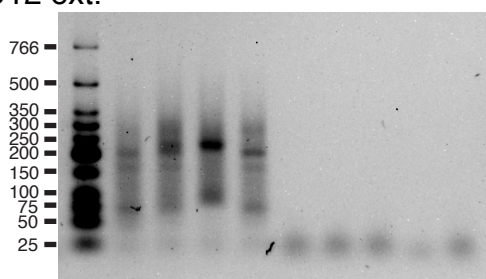

5S rRNA

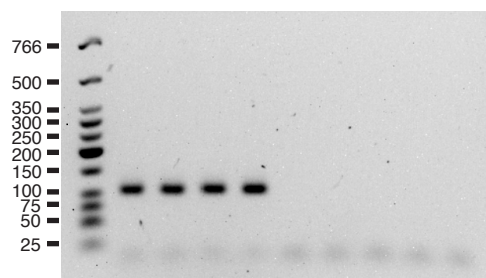

7SL RNA

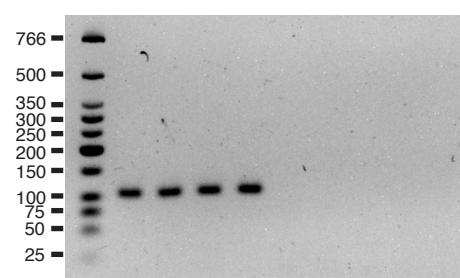

Figure 4F

RNU12 ext.

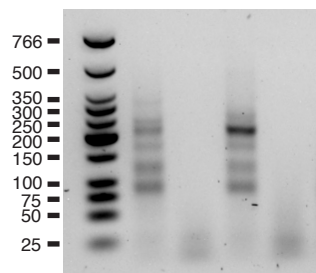

OAT

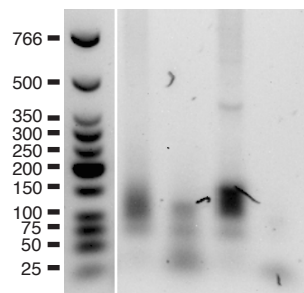

RPL12

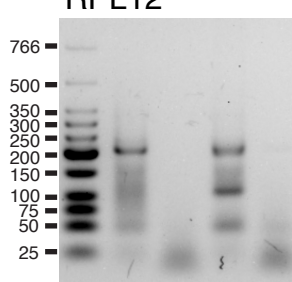

5S rRNA

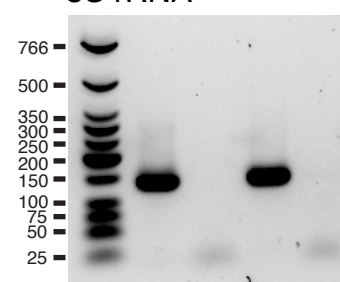

Supplement: Supplementary file 5 — Source Data for Figure 4 [file EMBJ-35-2179-s004.pdf]
